# Gate Modulation of Graphene-ZnO Nanowire Schottky Diode

**DOI:** 10.1038/srep10125

**Published:** 2015-05-06

**Authors:** Ren Liu, Xu-Chen You, Xue-Wen Fu, Fang Lin, Jie Meng, Da-Peng Yu, Zhi-Min Liao

**Affiliations:** 1 State Key Laboratory for Mesoscopic Physics, Department of Physics, Peking University, Beijing 100871, China; 2 Collaborative Innovation Center of Quantum Matter, Beijing, China

## Abstract

Graphene-semiconductor interface is important for the applications in electronic and optoelectronic devices. Here we report the modulation of the electric transport properties of graphene/ZnO nanowire Schottky diode by gate voltage (V_g_). The ideality factor of the graphene/ZnO nanowire Schottky diode is ~1.7, and the Schottky barrier height is ~0.28 eV without external V_g_. The Schottky barrier height is sensitive to V_g_ due to the variation of Fermi level of graphene. The barrier height increases quickly with sweeping V_g_ towards the negative value, while decreases slowly towards the positive V_g_. Our results are helpful to understand the fundamental mechanism of the electric transport in graphene-semiconductor Schottky diode.

Graphene, a typical two-dimensional (2D) material, due to the unique physical properties such as high carrier mobility and conductivity^1^, high optical transparency^2^ and mechanical flexibility^3^ *etc.*, has attracted great research interest for electronic and optoelectronic applications^4^. Especially the unique linear energy band dispersion and high conductivity make it suitable for the electrodes and the formation of Schottky barrier with various semiconductors^5^,^6^. Graphene-semiconductor Schottky junction has been widely investigated for applications in photodetectors^7^,^8^,^9^, solar cells^10^,^11^, and chemical and biological sensors^8^,^12^. Despite the recent progresses in graphene Schottky junctions with various semiconductors, it is crucial to tune the interface barrier to improve their performance in electronic and optoelectronic devices.

ZnO is a typical wide direct band gap (3.37 eV) semiconductor with large exciton binding energy (60 meV), which has been widely investigated for applications in optoelectronic devices^13^. Due to large surface-to-volume ratio, ZnO nanowires (NWs) exhibit highly susceptible photoelectric properties and have great potential in high sensitivity and fast optoelectronic sensors^14^. Recently, the graphene/ZnO NW junctions have been demonstrated for photodetectors and photovoltaic devices^7^,^15^. Nevertheless, the tuning of the Schottky barrier by external gate electric field is still elusive and important for the graphene/ZnO hetero-junction applications.

In this work, taking a combination of graphene and ZnO NW with respect to their unique properties, we manufactured graphene/ZnO NW Schottky diodes and carried out characterization of their electric transport properties. The ideality factor of the graphene/ZnO NW Schottky diode is ~1.7, and the Schottky barrier height is ~0.28 eV. Further gate controlling tests demonstrate that the Schottky barrier height is sensitive to the gate voltage, which mainly stems from the high sensitivity of Fermi level of graphene to the gate voltage. The barrier height increases quickly by applying negative gate voltage, while decreases slowly with increase of the positive gate voltage. Our results are helpful to understand the fundamental mechanism of the electric transport in graphene-semiconductor Schottky diode and may be useful for its application in nanoelectronics.

## Results

**Figure 1:**
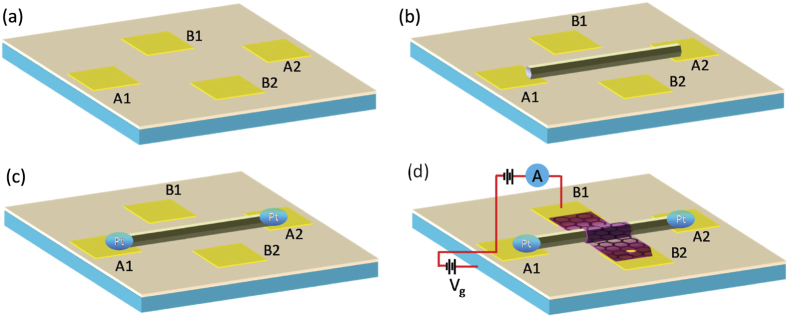
Schematic diagrams of the processes of fabricating graphene/ZnO nanowire Schottky diodes.

The vertical hybrid hetero-junction diodes of graphene/ZnO NW were fabricated *via* the processes as presented in Figure 1. First, four gold (Au) electrodes (two transverse ones and two longitudinal ones, thickness of 80 nm) were fabricated on a heavily doped Si substrate with a 300 nm SiO_2_ layer (see Fig. 1(a)). Second, an individual ZnO NW (diameter ~ 300 nm) was transferred onto a pair of Au electrodes (marked as A1 and A2) by micromanipulation under an optical microscope (see Fig. 1(b)). In order to obtain good Ohmic contact, the two ends of ZnO NW were further connected with the Au electrodes by Pt mends using focused ion beam induced metal deposition (see Fig. 1(c)). Then, monolayer graphene was transferred onto the ZnO NW and bridging another pair of Au electrodes (marked as B1 and B2) using a site-specific transfer-printing method (see Fig. 1(d))^16^,^17^,^18^. In this kind of vertical hybrid hetero-junction structure, the interface between graphene and ZnO NW will forms a Schottky barrier. As schematically shown in Fig. 1(d), the electric transport properties of ZnO NW, graphene and graphene/ZnO NW Schottky barrier can be characterized by electrical measurements using the electrode pairs of A1-A2, B1-B2 and A1-B1, respectively. The Si substrate severs as the back gate electrode.

**Figure 2:**
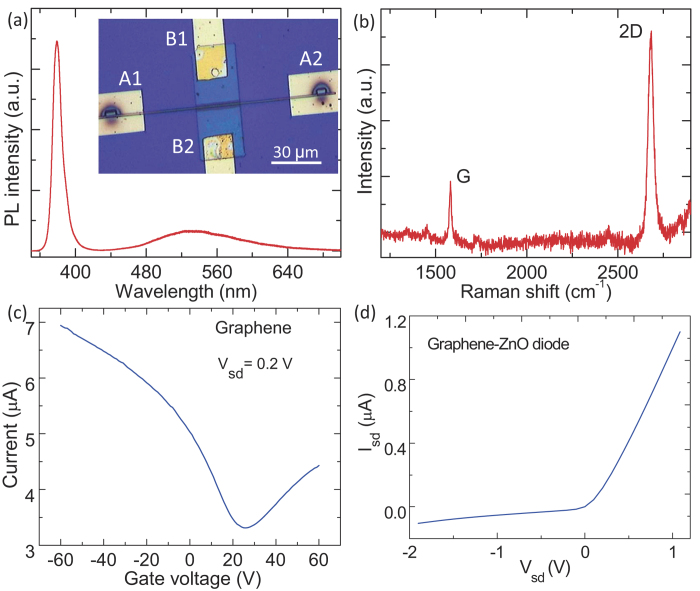
(**a**) PL spectrum of the ZnO NW in the graphene/ZnO NW Schottky diode. The inset figure is a typical optical image of the graphene/ZnO NW junction. (**b**) Raman spectrum of the graphene in the graphene/ZnO NW Schottky diode. (**c**) Source-drain current of the graphene (B1 and B2 electrodes) as a function of the back gate voltage. (**d**) *I*_*sd*_-*V*_*sd*_ characteristic of the graphene/ZnO NW Schottky diode (B1 and A1 electrodes).

The inset in Figure 2(a) shows a typical optical image of the fabricated graphene/ZnO NW Schottky diode. The black spots at the two ends of the ZnO NW are the deposited Pt metal, which form good Ohmic contact with ZnO^19^. Figure 2(a) presents the photoluminescence (PL) spectrum of the ZnO NW, which exhibits a very sharp and intensive near-band-edge emission peak centered at about 379.6 nm and a quite broad and weak green emission band, demonstrating the high crystal quality of the ZnO NW. The typical Raman spectrum of the chemical vapor deposition (CVD) graphene is shown in Fig. 2(b). The strong 2D peak (~2688 cm^−1^) and G peak (~1589 cm^−1^) can be clearly observed, while the defect related peak is quite weak. The Raman intensity ratio between the 2D mode and the G mode is ~3, indicating the nature of monolayer graphene^20^. Fig. 2(c) shows the source-drain current (I_sd_) of the monolayer graphene as a function of the back gate voltage (V_g_) as applying the source-drain voltage (V_sd_) of 0.2 V between the electrodes B1-B2. The I_sd_ of graphene can be changed by V_g_, suggesting that the Fermi energy level of graphene can be effectively tuned by V_g_. The current-voltage (I-V) curve of the graphene/ZnO NW hetero-junction is plotted in Fig. 2(d), as measured with B1 electrode connected to bias source and A1 electrode connected to ground. The nonlinear I-V curve shows typical rectifying behavior, demonstrating that Schottky barrier exists at the interface between the ZnO NW and graphene.

The formation of Schottky barrier at the interface between graphene and ZnO NW is due to the difference of work function between the two materials. The barrier height can be described as 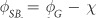 without considering the surface states, where 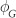 is the work function of graphene and χ is the electronic affinity of ZnO. Under forward bias, the current (*I*) passing through the Schottky barrier is determined by the thermionic emission of electrons, which can be written as^15^

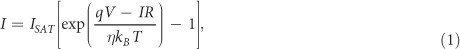


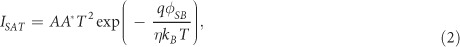
 where *I*_*SAT*_ is the saturation current, *q* is the elemental charge, *η* is the ideality factor 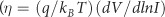 (here the ln is the natural logarithm function)), *R* is the series resistance, *k* is the Boltzmann constant, *T* is the absolute temperature, *ϕ*_*SB*_ is the Schottky barrier height, *A* is the interface area of the Schottky barrier, and A^*^ is the Richardson constant (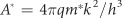) with the value of 32 A cm^−2^ K^−2^ for ZnO, and m^*^ is the effective mass of charge carriers (m^*^ = 0.27*m*_0_)^15^. Considering the effective contact area between graphene and ZnO NW *A* ~ 1 μm^2^ and the experimental temperature *T* ~ 300 K, according to Equations (1) and (2), the ideality factor of the diode can be deduced to be *η ~ *1.7. The barrier height without gate voltage can be deduced to be *ϕ*_*SB*_ ~ 0.28 eV. Since the work function for intrinsic graphene is about 4.6 eV and the electron affinity of ZnO NW is about 4.7 eV, the analysis is reasonable according to the experimental results.

**Figure 3:**
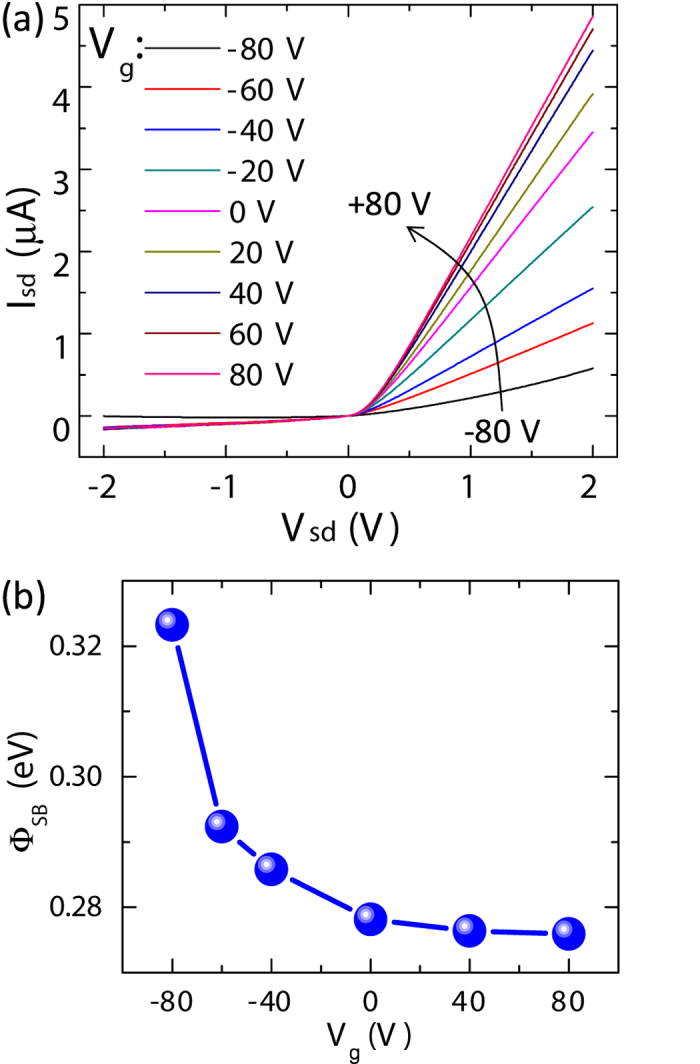
(**a**) The *I*_sd_-*V*_sd_ characteristics of the graphene/ZnO NW Schottky diode under different gate voltages (−80 ~ 80 V). (**b**) Variation of the Schottky barrier height of the graphene/ZnO NW junction as a function of gate voltage.

In order to further investigate the behavior of the Schottky barrier of the graphene/ZnO NW diode, the source-drain I_sd_-V_sd_ curves under different V_g_ were measured. The I_sd_-V_sd_ curves between electrodes B1 and A1 of the graphene/ZnO NW hetero-structure under different V_g_ are presented in Figure 3(a). As V_g_ sweeping from −80 V to 0 V, the I_sd_ at the same positive V_sd_ increases quickly, and then increase slowly with V_g_ further increasing from 0 V to 80 V. The I_sd_-V_sd_ curves at the negative V_sd_ side exhibit no apparent variation with V_g_. Using Equations (1) and (2), we can deduce the Schottky barrier height from each I_sd_-V_sd_ curve under different V_g_. The variation of Schottky barrier height as a function of V_g_ was plotted in Fig. 3(b). The barrier height increases quickly as varying V_g_ from 0 to −80 V, while decreases slowly as varying V_g_ from 0 to 80 V. This gate voltage modulation of the Schottky barrier height in graphene/ZnO NW hetero-junction is attributed to the linear dispersion relationship of the energy band in graphene, which results in the high sensitivity of Fermi level of graphene to V_g_.

## Discussion

**Figure 4:**
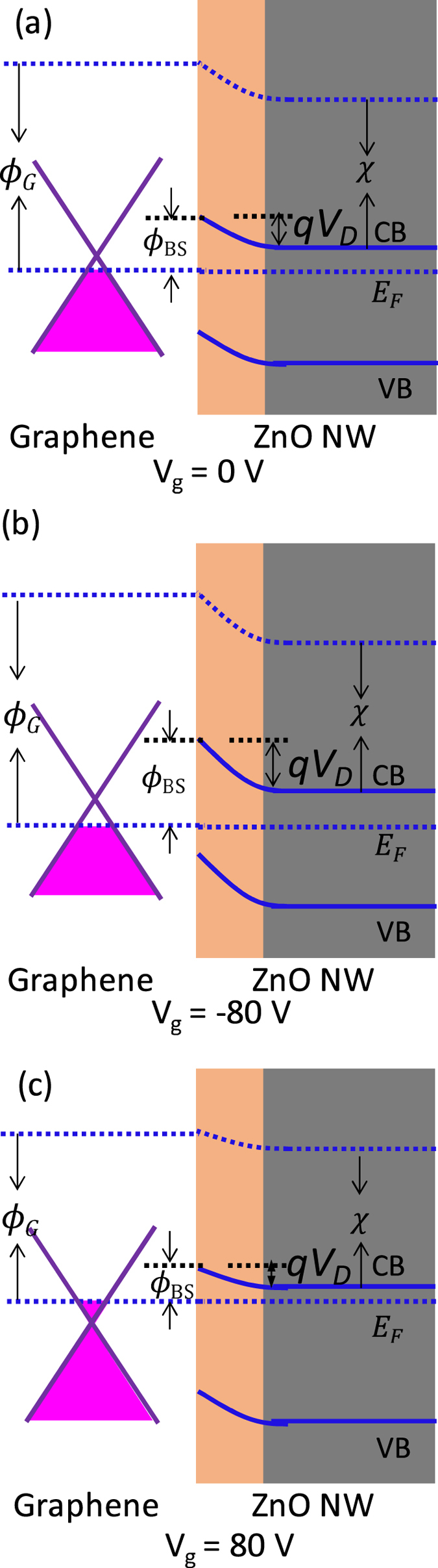
Schematic diagrams of the energy bands at the interface of Graphene/ZnO nanowire at different gate voltages with (**a**) 0 V, (**b**) −80 V, and (**c**) 80 V.

The underlying mechanism of the gate tuning of graphene/ZnO NW Schottky diode can be understood according to the energy bands of graphene and ZnO NW contact interface, as shown in Fig. 4. Because the graphene is p-type doped (Fig. 2(c)), the Fermi level of the graphene is below the Dirac point without applying V_g_. The electron transfer from ZnO to graphene results in the energy band bending near the ZnO NW surface and the formation of a depletion region. The Schottky barrier height is 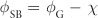 (see Figure 4(a)). As varying V_g_ from 0 to −80 V, the *ϕ*_*SB*_ of graphene continuously increases, leading to the notable increases of *ϕ*_*SB*_ (see Fig. 4(b)). As varying V_g_ from 0 to 80 V, both the Fermi levels of graphene and ZnO NW are lifting, while the shift of Fermi level of graphene is larger than that of ZnO because the linear energy band of graphene and the parabolic energy band of ZnO. Moreover, the Fermi level of ZnO may also somehow be pinned by the surface states due to its n-type conduction and the relatively high carrier density under positive V_g_, resulting in a small variation of the Schottky barrier height, as shown in Fig. 4(c).

In conclusion, we fabricated graphene/ZnO NW Schottky diodes and studied the electric transport properties. The ideality factor of the graphene/ZnO NW Schottky diode is ~1.7 and the Schottky barrier height is ~0.28 eV. The Schottky barrier height of the graphene/ZnO NW diode is sensitive to the gate voltage, which is ascribed to the gate tuning of Fermi level of graphene. The barrier height increases quickly with applying gate voltage from 0 to −80 V, while decreases slowly with increasing the gate voltage from 0 to 80 V. Our results are helpful to tune the graphene-ZnO NW Schottky diode and may have potential applications in nanoelectronics.

## Methods

Here, the high quality monolayer graphene was grown on a 25 μm thick copper foil by CVD method as described elsewhere^16^,^17^. Because the Schottky barrier is also highly influenced by the interface properties, a clean graphene/ZnO interface is necessary. The graphene layer was rained many times in 60 °C de-ionized water after it was exfoliated from the Cu foil using PMMA as carrier film. The graphene/PMMA was then taken out from the de-ionized water and processed as a suspended film. The adsorptions, such as water molecules, were further removed via baking before the graphene was transferred onto the ZnO nanowire. The ZnO NWs were synthesized *via* a simple CVD method, which grew along the [0001] zone axis with hexagonal cross-section^18^. The Raman spectrum of graphene and the PL spectrum of individual ZnO NW were measured by micro-zone confocal Raman spectroscope (Renishaw inVia micro-Raman system). For the Raman measurement of graphene, a 514 nm laser was used for excitation. The PL spectrum of ZnO NW was measured with the excitation of He-Cd laser with wavelength of 325 nm. The electric transport properties of graphene/ZnO NW hetero-junction were characterized by Keithley 4200 source-meter units. All of the measurements were carried out at room temperature in ambient.

## Author Contributions

Z.M.L. conceived and designed the study. R.L., X.W.F., F.L. and J.M. performed the experiments. D.P.Y. gave scientific advice. Z.M.L., X.C.Y. and X.W.F. wrote the manuscript. All authors contributed to discussion and reviewed the manuscript.

## Additional Information

**How to cite this article**: Liu, R. *et al*. Gate Modulation of Graphene-ZnO Nanowire Schottky Diode. *Sci. Rep.* **5**, 10125; doi: 10.1038/srep10125 (2015).
